# Genome-Wide Identification and Evolutionary Analysis of *Gossypium* YTH Domain-Containing RNA-Binding Protein Family and the Role of GhYTH8 in Response to Drought Stress

**DOI:** 10.3390/plants12051198

**Published:** 2023-03-06

**Authors:** Wei Hao, Weipeng Wang, Xiangfen Xiao, Jialiang Sun, Bingjie Wu, Yuping Zhao, Shuaishuai Pei, Wenjia Fan, Dongbei Xu, Tengfei Qin

**Affiliations:** 1 College of Medical Technology, Beihua University, Jilin City 132013, China; 2 College of Agriculture, Liaocheng University, Liaocheng 252000, China; 3 Henan Collaborative Innovation Center of Modern Biological Breeding, Henan Institute of Science and Technology, Xinxiang 410700, China; 4 Shanghai Center for Plant Stress Biology, Shanghai Institutes for Biological Sciences, Chinese Academy of Sciences, Shanghai 201602, China; 5 College of Agronomy, Sichuan Agricultural University, Chengdu 625014, China

**Keywords:** *Gossypium*, drought stress, YTH domain-containing RNA-binding protein, gene family

## Abstract

YTH domain-containing proteins are one kind of RNA-binding protein involved in post-transcriptional regulation and play multiple roles in regulating the growth, development, and abiotic stress responses of plants. However, the YTH domain-containing RNA-binding protein family has not been previously studied in cotton. In this study, a total of 10, 11, 22, and 21 *YTH* genes were identified in *Gossypium arboreum*, *Gossypium raimondii*, *Gossypium barbadense*, and *Gossypium hirsutum*, respectively. These *Gossypium YTH* genes were categorized into three subgroups by phylogenetic analysis. The chromosomal distribution, synteny analysis, structures of *Gossypium YTH* genes, and the motifs of YTH proteins were analyzed. Furthermore, the *cis*-element of *GhYTH* genes promoter, miRNA targets of *GhYTH* genes, and subcellular localization of GhYTH8 and GhYTH16 were characterized. Expression patterns of *GhYTH* genes in different tissues, organs, and in response to different stresses were also analyzed. Moreover, functional verifications revealed that silencing *GhYTH8* attenuated the drought tolerance in the upland cotton TM-1 line. These findings provide useful clues for the functional and evolutionary analysis of *YTH* genes in cotton.

## 1. Introduction

The post-transcriptional regulation of gene expression is of great importance for various biological processes, such as plant growth and development, and environmental stress responses. Many processes are involved in post-transcriptional regulation, including pre-mRNA processing, polyadenylation, mRNA stability, and RNA transport [1,2]. These processes could be directly mediated by RNA-binding proteins (RBPs) through interacting with target RNA molecules or indirectly mediated by RBPs by regulating the function of other regulatory factors [3]. The study of RBPs in plants was slower than other organisms due to the lack of appropriate plant-derived in vitro systems for the research of post-transcriptional gene regulation. More than 200 RBPs were identified in *Arabidopsis thanliana*, and about 250 RBPs were identified in rice. Most of these identified RBPs are plant-specific and can act in plant-specific functions, such as the involvement in hormone responses and various environmental stresses [4,5].

YTH domain-containing proteins are one class of RBPs. In 1998, Imai Y et al. reported one member of the YTH-containing proteins in rats named YT521 [6]. YT521 was an RNA splicing-related protein that could interact with splicing factors in yeast two-hybrid and co-immunoprecipitation assays [7]. In 2002, Stoilov et al. reported the identification of a novel YTH domain (for YT521-B homology) in nuclear proteins. They also found that the YTH domain, containing 100–150 residues, was highly conserved and typical for the eukaryotes via homology searches. They also showed that the YTH domain was abundant in plants [8]. The first YTH domain-containing RNA-binding protein AtCPSF30 was identified in *A. thanliana*. AtCPSF30 is a part of the plant polyadenylation complex that could act as a processing endonuclease [9]. N6-Methyladenosine (m^6^A) is the most abundant and widespread modification in eukaryotic RNAs [10]. In recent years, many studies revealed that YTH proteins are involved in the regulation of the dynamic RNA N6-Methyladenosine (m^6^A) modification, which is essential for gene regulation and the maintenance of genome stability [11]. Five YTH proteins (YTHDC1–2 and YTHDF1–3) have been identified in humans. YTHDC1 and YTHDC2 act as m6A readers in the nucleus, while YTHDF1–3 act as cytoplasmic m6A readers [12]. With the rapid development of next-generation sequencing, genome-wide identification and evolutionary analysis of the *YTH* genes have been reported in some plant species, such as *Arabidopsis*, rice [13], apple [14], tomato [15], and wheat [16]. Thirteen YTH domain proteins were identified in tomato, including ECT1–11, CPSF30, and At4g11970 [17]. Arabidopsis ECT2, the homolog of YTHDFs, can bind to m6A-containing RNAs in vivo, suggesting that the YTH domain is also an m6A reading domain in plant [18]. Tomato SlYTH1, a putative RNA m6A reader, plays important roles in seed germination, plant growth and development, and fruit morphology [19].

Some studies have shown that the YTH domain-containing RNA-binding proteins are involved in responses to abiotic stress in plants. Li et al. found that many *Arabidopsis* and rice *YTH* genes might participate in responses to abiotic stresses, such as the cold, drought, salt, and heat stress, via analyzing the publicly available microarray data [13]. Wang et al. [14] discovered that *YTH* genes showed various level responses under different stress treatments in apples. They found that the *YTH* genes were more sensitive to chilling and H_2_O_2_ conditions than ABA, heat, drought, and salt conditions [14]. Overexpression of *MhYTP1* and *MhYTP2* from apples enhanced transgenic *Arabidopsis* plants resistance to salinity and drought [20]. The expression levels of some *YTH* genes in cucumbers were altered under different kinds of stresses, including salt, drought, and cold treatments [21].

Cotton, one of the most important cash crops, is the main source of natural textile fiber worldwide. The genome sequence of different cotton species has been released successively, such as the ancestral diploids, *G. arboreum* and *G. raimondii*, and the tetraploids, *G. hirsutum* and *G. barbadense*. Therefore, it becomes possible to explore gene families and their putative functions in various cotton species. Up to now, YTH domain-containing RNA-binding protein family members have not been reported in cotton, especially the ones that participate in the environmental stress tolerance of cotton.

In the present study, we identified the YTH domain-containing RNA-binding proteins in four cotton species and analyzed their physiochemical properties. We investigated their phylogenetic relationship, gene structure, and conserved motifs in cotton. Moreover, we analyzed the expression patterns of *YTH* genes in different tissues and the response of *YTH* genes to different stresses, including the cold, heat, drought, and salt. In addition, we predicted the miRNA targets of *GhYTH* genes to reveal the relationship between microRNA (miRNA) and *YTH* family genes in *G. hirsutum*. Furthermore, we carried out subcellular localization of GhYTH8 and GhYTH16. Additionally, we used VIGS technology to verify *GhYTH8* genes positively regulating drought stress tolerance in cotton. Our results lay the foundation for functional and evolutionary studies of *YTH* genes in plants.

## 2. Results

### 2.1. YTH Gene Family in Four Gossypium Species

The whole protein sequences of two diploid species, *G. arboretum* and *G. raimondii*, and two tetraploid species, *G. barbadense* and *G. hirsutum*, to identify cotton *YTH* genes. The identified *YTH* genes were further confirmed via conserved YTH domain searches. In total, we identified 64 *YTH* genes from the 4 cotton species, including 10 *GaYTHs*, 11 *GrYTHs*, 22 *GbYTHs*, and 21 *GhYTHs* (Table 1). The amino acid residue length of YTH proteins in cotton varied from 572 to 677 aa in *G. arboreum*, 572 to 670 aa in *G. raimondii*, 559 to 689 aa in *G. barbadense*, and 568 to 720 aa in *G. hirsutum*. The isoelectric points (pI) ranged from 5.32 to 7.22 in *G. arboreum*, 5.14 to 6.9 in *G. raimondii*, 5.02 to 8.48 in *G. barbadense*, and 5.2 to 8.25 in *G. hirsutum*. The molecular weight was distributed approximately from 63.5 to 74.3 kDa, 63.3 to 74.0 kDa, 62.1 to 76.2 kDa, and 62.3 to 81.1 kDa in *G. arboreum*, *G. raimondii*, *G. barbadense*, and *G. hirsutum*, respectively.

### 2.2. Phylogenetic Analysis of YTH Genes

**Figure 1:**
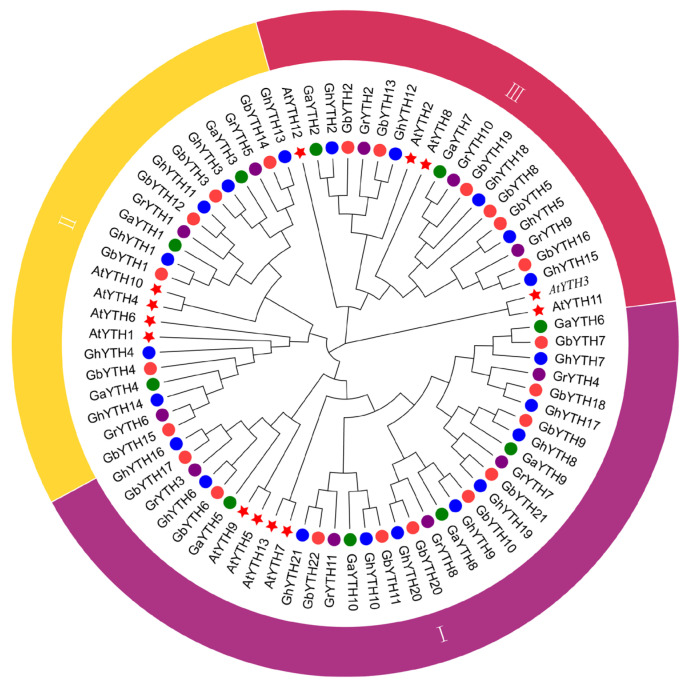
Phylogenetic analysis of *YTH* gene family in *Gossypium* and *Arabidopsis*. The phylogenetic tree resolved *YTH* genes into three groups. The phylogenetic ML tree was constructed using the amino acid sequence with a 1000 bootstrap value with MEGA-X software (version 7.0). *YTH* genes were categorized into threegroups from Group I to Group III. Ga, *Gossypium arboretum*; Gr, *Gossypium raimondii*; Gh, *Gossypium hirsutum*; Gb, *Gossypium barbadense*; At, *Arabidopsis thaliana*.

To study the evolutionary relationships among 64 cotton *YTH* genes, protein sequences from the 4 cotton species and *Arabidopsis* were used to build a phylogenetic tree using the ML method. The cotton *YTH* genes were categorized into three subgroups from Group I to Group III. Genes that were divided into the same group were more closely related. Group I to Group III contains 30, 18, and 16 cotton *YTH* genes, respectively. There were 5, 3, and 2 *GaYTHs* contained in Group I to Group III in *G. arboretum*, respectively, and 5, 3, and 3 *GrYTHs* in *G. raimondii*. While 10, 6, and 6 *GbYTHs* were classified into Group I to Group III, respectively, in *G. barbadense*, and 10, 6, and 5 *GhYTHs*, in *G. hirsutum* (Figure 1). The phylogenetic tree displayed that *G. hirsutum* and *G. barbadense* tend to be assigned to the same branch, suggesting a closer relationship between these two species.

### 2.3. Motifs and Gene Structure Analysis

**Figure 2:**
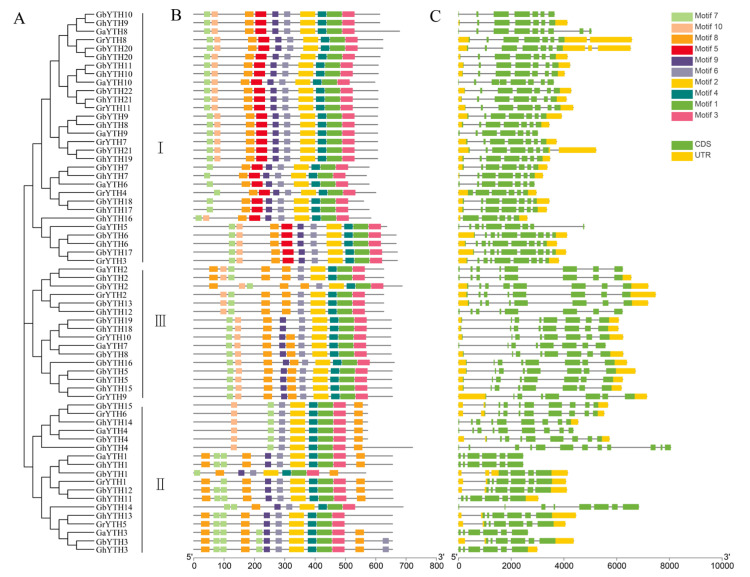
Phylogenetic tree, gene structure, and conserved protein motifs of *YTH* genes in 4 cotton species. (**A**) Phylogenetic tree of 4 cotton species. (**B**) Protein motifs in *Gossypium YTHs*. Each colored box indicates a specific motif. (**C**) The exon–intron structure of cotton *YTH* genes. The yellow boxes represent untranslated regions (UTRs), the green boxes indicate exons, and the grey lines indicate introns.

To explore the evolutionary patterns and classification of *YTHs* in cotton, the unrooted phylogenetic tree was constructed. The resultant phylogenetic tree of four cotton species divided the YTH proteins into three main groups (Groups I–III) (Figure 2A). Based on the phylogenetic result, we analyzed the motifs and the sequence structure of the identified *YTHs*. To explore the conserved motif organization in YTH proteins, the MEME program was used for the conserved motif analysis. A total of 10 motifs were identified in the YTH proteins. Motifs 1–4 and 6–8 were found in all YTH proteins. Motifs 1–9 were identified in Group I, and especially Motif 5 was only found in Group I (Figure 2B). Furthermore, in most of the YTH proteins, the YTH domain is the only recognizable module that is in line with those of other species [13]. In addition, dnaA superfamily domain and PHA03377 superfamily domain were also found in several YTH proteins. The dnaA superfamily domain was only found in Group I, while the PHA03377 superfamily domain was only found in Group III (Supplementary Figure S1).

To reveal the consistency of the exon–intron pattern in the phylogenetic groups, we performed the gene structure analysis on the cotton *YTH* genes. Our data revealed that the number of exons varied from 6~9 in *G. arboreum*, 6~10 in *G. raimondii*, 6~11 in *G. barbadense*, and 6~12 in *G. hirsutum* in *YTH* gene family. The exons and introns arrangement uncovered the evolutionary relationships between different gene family members. The *YTH* genes that have a similar exon–intron pattern tend to be clustered into a similar branch. Particularly, in Group III, most of the exon numbers of the *YTH* genes were 8, except for *GhYTH18* (Figure 2C, Table S1). Our results showed that the closely associated genes are more structurally similar and differ in the length of intron and exons.

### 2.4. Chromosome Distribution and Synteny Analysis of YTH Genes

**Figure 3:**
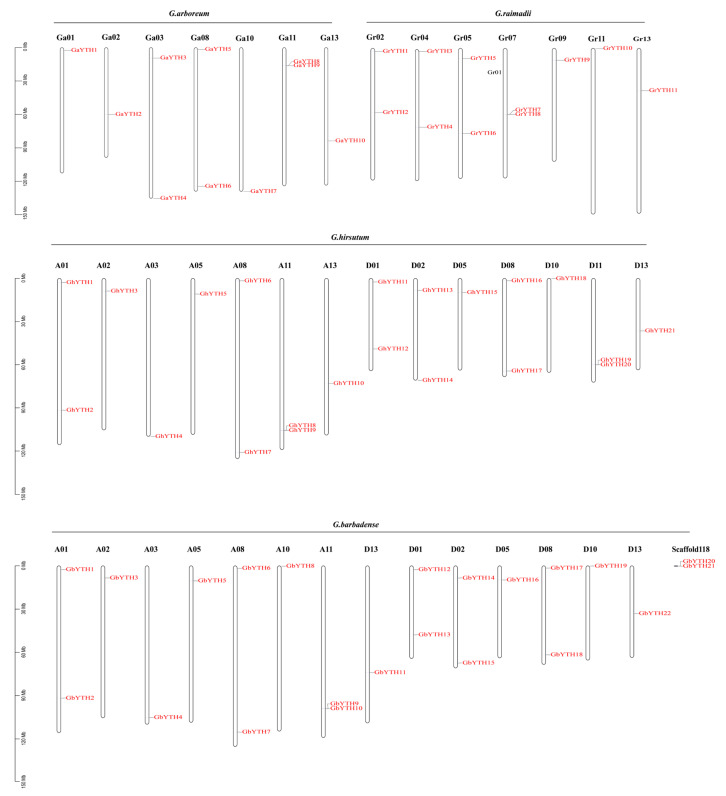
Distribution of the *YTH* genes on chromosomes of *G. arboretum*, *G. raimondii*, *G. hirsutum*, and *G. barbadense*.

The chromosomal distribution of *YTH* genes was identified according to the genome annotation file. *YTH* genes were distributed on chromosomes 1–5, 9–11, and 13. *GaYTH* genes were localized across chromosomes 1–3, 8, 10, 11, and 13. *GrYTH* genes were localized across chromosomes 2, 4, 5, 7, 9, 11, and 13. *GhYTHs* were distributed on At chromosomes 1–3, 5, 8, 11, 13, and Dt chromosomes 1, 2, 5, 8, 10, 11, and 13 with 10 and 11 genes, respectively. *GbYTHs* were located on At chromosomes 1–3, 5, 8, 10, 11, 13, and Dt chromosomes 1, 2, 5, 8, 10, 13 with 11 and 9 genes, respectively. Another two *GbYTHs* (*GbYPH20* and *GbYPH21*) were identified on unanchored scaffolds. Results showed that the chromosome distribution pattern between *GbYTHs* and *GhYTHs* was similar (Figure 3). 

**Figure 4:**
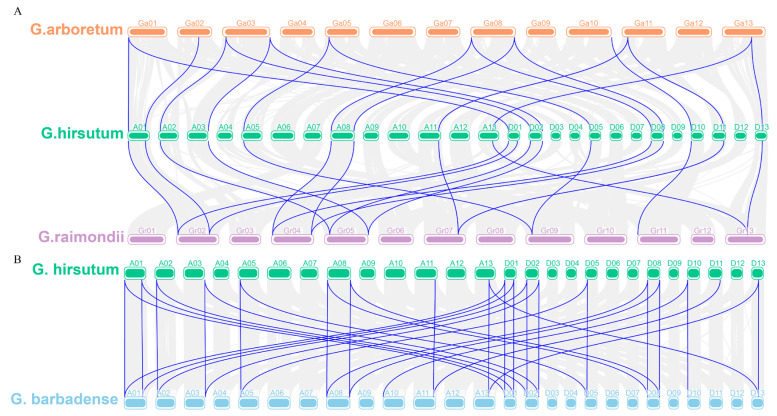
The synteny analysis of *YTH* genes in four cotton species. (**A**) The collinear relationship between *Gossypium arboreum*, *Gossypium raimondii*, and *Gossypium hirsutum*. (**B**) The collinear relationship between *Gossypium hirsutum* and *Gossypium barbadense*. Blue lines represent syntenic *YTH* gene pairs. Grey lines indicate collinear block.

To further explore the phylogenetic mechanisms of the cotton *YTH* gene family, we performed the synteny analysis of *YTH* genes between *G. hirsutum* and the three other representative cotton species. A total of 32 *GhYTH* genes showed syntenic relationship with those in *G. barbadense*, followed by *G. raimondii* (19) and *G. arboretum* (18). Some *GhYTH* genes were found to have at least three syntenic gene pairs, suggesting that these genes may have played an essential role during evolution (Figure 4).

To better reveal *G. hirsutum* replication events, we identified all 32 homologous genes in *G. hirsutum* and compared them with those in *G. barbadense*. We calculated the levels of Ka and differences in Ks (Table S2). Ka/Ks = 1.0 suggested pseudogenes caused by neutral selection, Ka/Ks < 1 implied purifying selection, and Ka/Ks > 1 denoted positive selection and accelerated evolution. The Ka/Ks ratios of most duplicate gene pairs were less than one (Table S2), indicating that *YTH* gene purification and positive selection existed during cotton evolution.

### 2.5. Cis-Regulatory Element Analysis of YTH Genes

**Figure 5:**
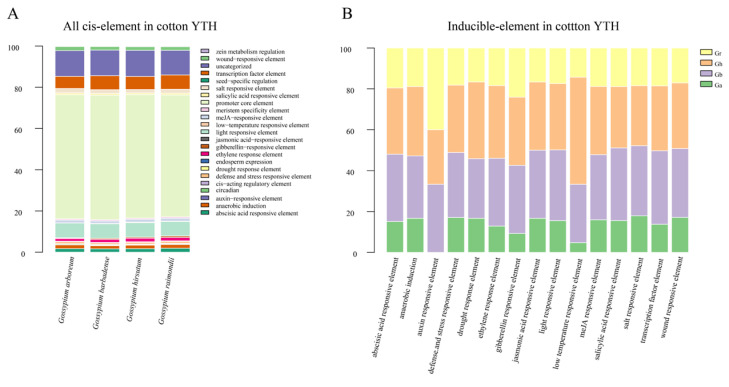
*Cis*-element analysis of *YTH* genes in 4 cotton species. (**A**) *Cis*-elements in the promoters of *YTHs*. (**B**) Inducible elements in the promoters of *YTHs*.

*Cis*-regulatory elements play important roles in gene expression regulation [22,23]. Understanding the *cis*-elements involved in the regulation of *YTH* genes will facilitate the study of the regulation mechanism and the putative functions of these *YTH* genes. The 2000 bp region upstream of the start codon of each *YTH* gene from 4 cotton species was extracted as the promoter region. The *cis*-acting elements, including salt responsive element, ethylene response element, and abscisic acid responsive element, were identified in the promoters of *YTH* genes of four cotton species. The number of these *cis*-acting elements in different species was basically consistent (Figure 5A). Inducible promoters can respond immediately to stimulus signals and be involved in the related gene expression regulation under particular environmental and stimulus conditions. Furthermore, we identified a total of 15 inducible promoters of *YTH* genes in cotton (Figure 5B).

### 2.6. Prediction of YTH Genes Targeted by MiRNAs in G. Hirsutum

**Figure 6:**
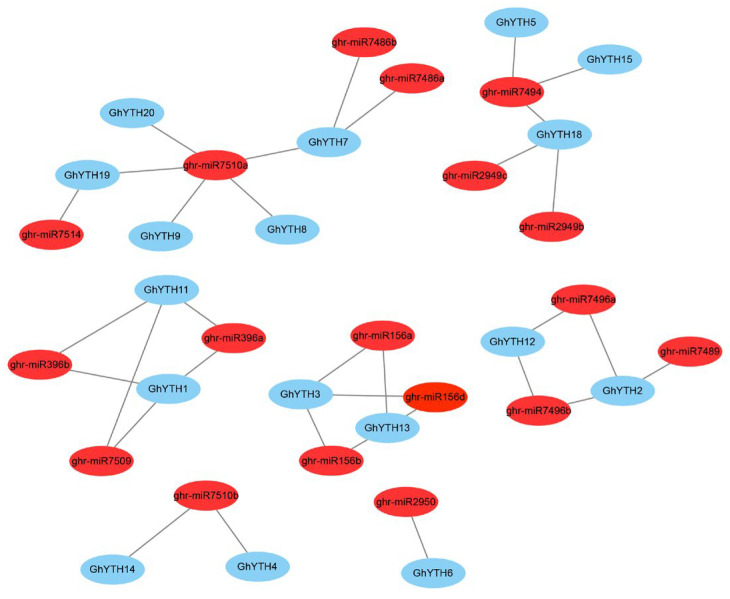
miRNA targets of *YTH* genes in *G. hirsutum*. The red circles represent miRNAs. The blue circles indicate *GhYTH* genes.

Unlike *cis*-regulatory elements, the miRNAs were considered transacting factors targeting the mRNA. The relationships between the miRNAs and *YTHs* in cotton were predicted in the psRNATarget database (Table S3). In total, 18 miRNA targets of *GhYTH* genes were identified. Among 21 identified *GhYTHs*, 17 of them were found to be targeted by miRNAs, except for *GhYTH10*, *GhYTH16*, *GhYTH17*, and *GhYTH21*. *GhYTH1*, *GhYTH2*, *GhYTH3*, *GhYTH7*, *GhYTH13*, and *GhYTH18* were targeted by three miRNAs, respectively. *GhYTH12* and *GhYTH19* were targeted by two miRNAs, respectively. Each of the other nine *GhYTHs* was only targeted by only one miRNA. Almost all miRNAs targeted a single or two *GhYTHs*. ghr-7510R and ghr-7494 targeted five and three *GhYTHs*, respectively (Figure 6).

### 2.7. Tissue Expression Patterns of YTH Genes in G. hirsutum

**Figure 7:**
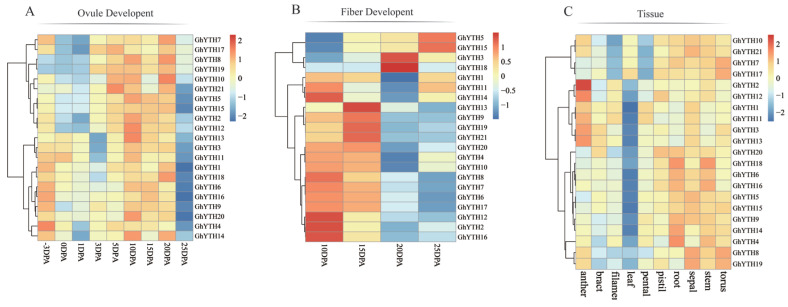
Expression patterns of *GhYTH* genes in different tissues. (**A**) Expression patterns of *GhYTHs* in ovule (−3, 0, 1, 3, 5, 10, 15, 20, and 25 DPA). (**B**) Expression patterns of *GhYTHs* in fiber (10, 15, 20, and 25 DPA). (**C**) Expression patterns of *GhYTHs* in different organs (torus, stem, sepal, root, pistil, petal, leaf, fila-ment, bract, and anther).

Gene expression patterns are closely related to their functions. To explore the spatial–temporal expression variations in *YTH* genes across various anatomical tissues, we analyzed the transcriptome data of *G. hirsutum* during the development of ovule and fiber (Table S4). In ovules, the expression levels of *GhYTH2/5/8/10/15/19/21* were higher from 5 DPA to 20 DPA (Figure 7A). During fiber development, the expression levels of *GhYTH5* and *GhYTH15* increased substantially from 10 DPA to 25 DPA. The expression levels of *GhYTH2/12/14/16*, *GhYTH13*, and *GhYTH3/18* were relatively high at 10 DPA, 15 DPA, and 20 DPA, respectively. The expression of *GhYTH4/6/7/8/10/12/17* was relatively high at 10 DPA and 15 DPA (Figure 7B). The results indicated that each *GhYTH* gene might have a specific function at different time points, including the initiation and elongation stages of cotton fibers.

**Figure 8:**
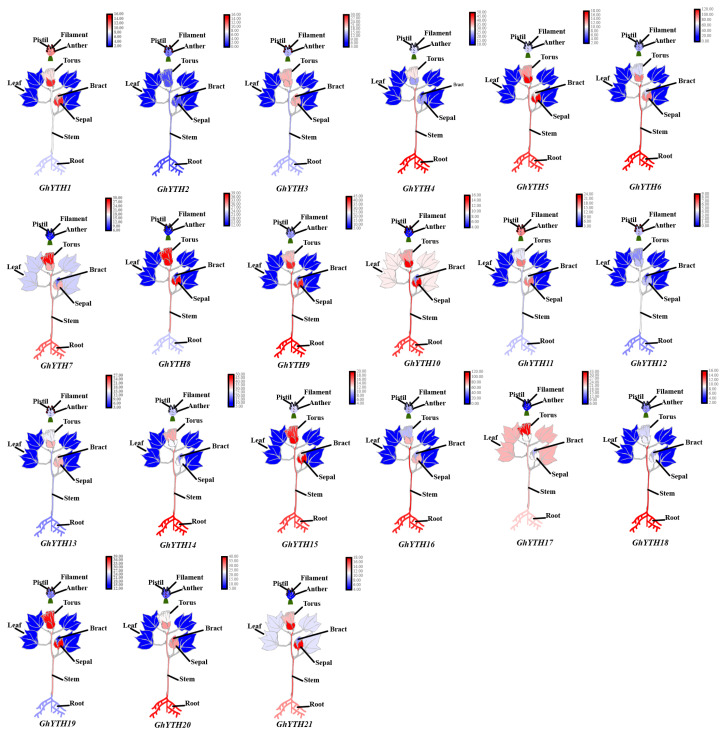
Expression patterns of *GhYTHs* in different organs (torus, stem, sepal, root, pistil, petal, leaf, filament, bract, and anther).

In addition, the expression profiles of *GhYTH* genes from ovule, fiber, torus, stem, sepal, root, pistil, petal, leaf, filament, bract, and anther were also studied. *GhYTH* genes displayed dynamic expression profiles in these tissues and organs (Table S4). Briefly, the expression of *GhYTHs* was relatively low in leaves, filaments, petals, and bracts. *GhYTH2/3/12/13* in anther, *GhYTH4/14/18* in root, *GhYTH8/19* in sepal, *GhYTH16/18* in stem, and *GhYTH7/8/17/19* in torus were highly expressed (Figure 7C and Figure 8). The results showed that *GhYTH* genes might be involved in the growth and development processes of different organs in cotton.

### 2.8. Expression Patterns of YTH Genes in G. hirsutum under Stress Conditions

**Figure 9:**
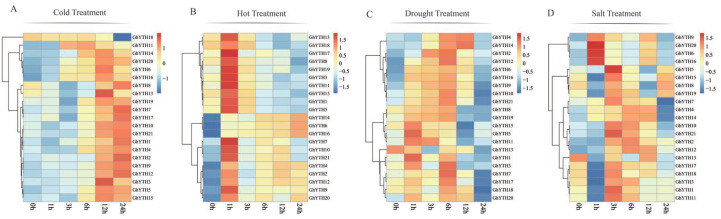
Expression patterns of *GhYTH* genes under different abiotic stresses. (**A**) Expression patterns of *GhYTHs* under cold stress (0, 1, 3, 6, 12, and 24 h). (**B**) Expression patterns of *GhYTHs* under hot stress (0, 1, 3, 6, 12, and 24 h). (**C**) Expression patterns of *GhYTHs* under drought stress (0, 1, 3, 6, 12, and 24 h). (**D**) Expression patterns of *GhYTHs* under salt stress (0, 1, 3, 6, 12, and 24 h).

We analyzed the transcriptome data of *G. hirsutum* to uncover the *GhYTH* participation in response to cold, hot, salt, drought, and salt stress conditions (Table S5). Under cold stress, the expression levels of *GhYTH11/18* reached a peak at 6 h after treatment and then decreased, the expression of *GhYTH3/5/6/13/14/15/16/19/20* peaked at 12 h after treatment then decreased, and the expression of *GhYTH1/2/4/7/8/9/10/12/17/21* was peaked at 24 h post-treatment (Figure 9A). Under hot stress, the expression of *GhYTH* genes peaked at 1 h and then decreased except for *GhYTH6/12/14/16*. However, the expression trend of *GhYTH6/14/16* continuously increased and peaked at 24 h post treatment (Figure 9B). Under drought stress, *GhYTH* genes exhibited higher expression in the first 6 h after treatment and then showed decreased expression except for *GhYTH1/4/5/11/13/14/15/20.* The expression level of *GhYTH2/6/8/9/10/12/16/19* gradually increased post-drought treatment, indicating their possible roles in response to drought stress. (Figure 9C). Under salt stress, *GhYTH6/16/20* were highly expressed at 1 h post-treatment (Figure 9D). In brief, our data showed that the expression level of most *YTH* genes was induced in response to multiple stresses.

### 2.9. Subcellular Localization of GhYTH8 and GhYTH16

**Figure 10:**
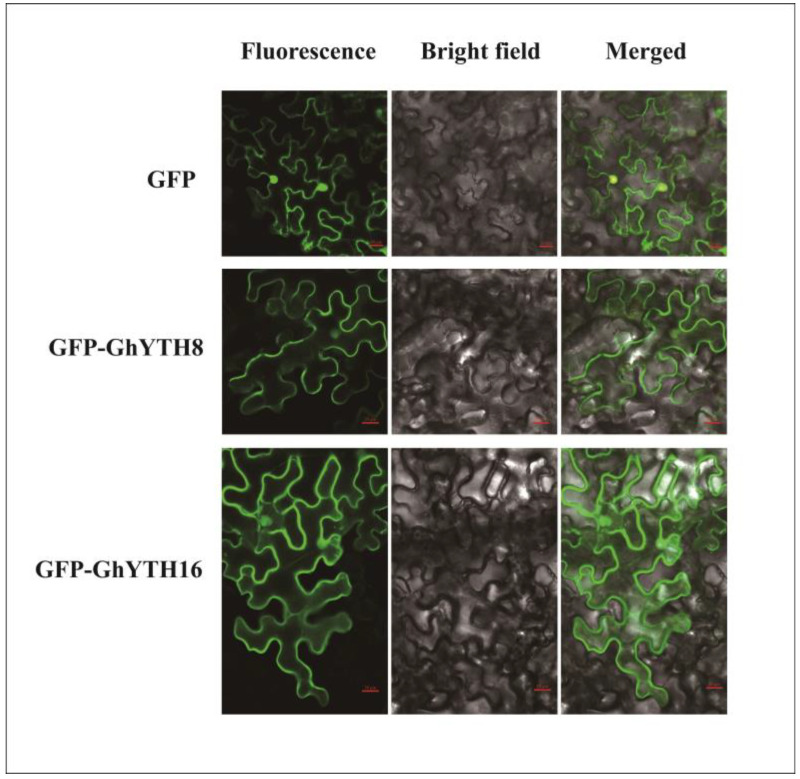
Subcellular analysis of GhYTH8 and GhYTH16 proteins. The GhYTH8::GFP, GhYTH16::GFP, and GFP alone were transiently expressed via agroinfiltration using *N. benthamiana* leaves. Green fluorescence was observed with confocal laser microscopy (left). The same cells were also observed by transmission microscopy, and both images were merged (from the middle to the right).

To investigate the subcellular localization of the GhYTH proteins, we fused the coding sequences of two of the GhYTH proteins (GhYTH8 and GhYTH16) that may be involved in the drought stress response to the GFP vector and transiently expressed in expanded leaves of *N. benthamiana*. Transient expression assays revealed that GFP signals in the GFP-YTH8 and GFP-GhYTH16 expressed in *N. benthamiana* leaves were observed in the whole cells, similar to the pattern observed for GFP alone (Figure 10). These results suggested that the GhYTH8 and GhYTH16 proteins do not localize in specific compartments and are likely targeted ubiquitously in cells, which indicates their functional diversity and complexity.

### 2.10. Functional Verification of GhYTH8 in Cotton under Drought Stress

**Figure 11:**
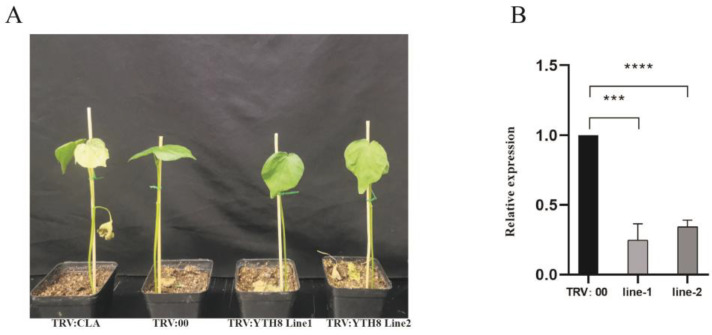
Functional verification of *GhYTH8* under drought stress. (**A**) Whole plant phenotypes of gene-silenced plants. The plant with CLA-silencing, the negative control plant, and two plants with GhYTH-silencing are displayed from left to right. (**B**) The expression level of *GhYTH* in empty control and VIGS plants under drought stress. *** and **** indicate the significant differences between the TRV::00 and TRV:GhYTH8 lines determined by Student’s t-test.

To further explore the role of *YTH* genes that may be involved in the drought stress response according to their expression levels under drought stress, we performed a VIGS assay based on the full-length sequence of GhYTH8 in TM-1. The success of the VIGS experiment was verified by the albinism of positive control leaves and the detection of silencing efficiency. When the cotton grew to the three-leaf stage, the cotton infected by pYL156:00 (TRV:00) was used as the control, and the cotton plants were kept without water to simulate drought conditions. After 14 days of drought treatment, we can observe significant differences of the phenotypes of leaves. Compared with the control, the leaves of pYL156:GhYTH8 (TRV:GhYTH8)-infected plants under drought conditions wilted obviously (Figure 11A). The expression level of *GhYTH8* was significantly lower in the silenced plants than in the control (TRV:00) (Figure 11B). Our data demonstrated that *GhYTH8-*silenced plants were more sensitive to drought stress, suggesting the functional roles of YTH proteins in the stress response of cotton.

## 3. Discussion

The YTH domain-containing RBP has been recognized in eukaryotes for more than 20 years. Nevertheless, polyploidization events increase the complexity and make it harder to recognize the role of a gene family. Cotton is an important economic crop around the world, which provides a natural fiber source for the textile industry. Genome and transcriptome sequencing have facilitated the understanding of gene families in cotton. However, the identification of *YTH* genes and their biological functions in cotton remained elusive. In this present study, a total of 64 *YTH* genes (10 *GaYTHs*, 11 *GrYTHs*, 22 *GbYTHs*, and 21 *GhYTHs*) were identified and characterized in four cotton species at the genome-wide scale. The genome size of *G. arboreum* (1746 Mb) is almost twice of *G. raimondii* (885 Mb) [24]. The genome size of *G. barbadense* (~2.22 Gb) is almost the same as that of *G. hirsutum* (~2.30 Gb) [25]. Previous studies reported that 13, 12, 9, 15, and 5 members of the *YTH* gene family were identified in *Arabidopsis*, rice, tomato, apple, and cucumber, respectively [21]. Our results showed that the number of *YTH* genes is not correlated with the size of the genomes.

The m^6^A modification is essential for modulating gene expression at the post-transcriptional level. To perform its regulatory function, the m^6^A modification complex needs to recruit reader proteins [26]. YTH family members have been shown to act as m^6^A reader proteins [27]. In humans, five YTH proteins, YTHDC1, YTHDC2, YTHDF1, YTHDF2, and YTHDF3, were identified. [28]. Studies have shown that a conserved mechanism was applied by YTHDFs and YTHDCs to identify m^6^A [29]. In this study, 64 *YTH* genes were clustered into three groups. *Arabidopsis YTH* genes *AtYTH7/9/13/5* (also named *AtDF1A-AtDF4A*), *AtYTH10/4/6/1* (also named *AtDF1C-AtDF4C*), *AtYTH8/2/12* (also named *AtDF1B-AtDF1B*) were clustered into Groups I-III, respectively. It is worth noting that although *AtYTH3* (*AtDC1A*) and *AtYTH11* (*AtDC1B*) were grouped into Group III, no other cotton *YTH* genes were in the same branch with these two *Arabidopsis YTHDC* genes. Furthermore, YTH1 superfamily domains were identified in all three common wheat YTHDC proteins [16]. However, no YTH1 superfamily domains have been found in cotton YTH proteins in this study, suggesting no *YTHDC* gene exists in cotton species. *G. barbadense* and *G. hirsutum* are allotetraploids that originated from the hybridization among A-genome-like ancestral African species and D-genome-like American species [30]. Ancestral diploids, *G. arboreum* and *G. raimondii*, contributed to the At and Dt subgenomes for both *G. hirsutum* and *G. barbadense*, respectively, through genome-wide duplication events [25,31]. *YTHs* located on At subgenomes in both *G. barbadense* (*GbYTH1*- *GbYTH11*) and *G. hirsutum* (*GhYTH1*- *GhYTH10*) tend to be clustered with *YTHs* in *G. arboreum* (A genome). Similarly, *YTHs* located on Dt subgenomes in both *G. barbadense* (*GbYTH12*- *GbYTH22*) and *G. hirsutum* (*GhYTH11*- *GhYTH21*) tend to be clustered with *YTHs* in *G. raimondii* (D genome). Our findings also confirmed the sources of At subgenomes and Dt subgenomes in two tetraploid species. *YTH* genes of *G. hirsutum* and *G. barbadense* tend to be clustered in the same branch. Our results indicated that the genetic relationship between two allotetraploids was closer than that between allotetraploids and diploids. It might be due to the coevolution of *G. hirsutum* and *G. barbadense*.

*GaYTH* and *GrYTH* genes are dispersed across 7 out of 13 chromosomes. Moreover, *GbYTH* and *GhYTH* genes are distributed on 7 out of 13 chromosomes across both subgenomes. *YTH* genes showed an uneven distribution on cotton chromosomes. This chromosomal distribution pattern of *YTH* genes was also found in Arabidopsis, rice, and cucumber [13,21]. It is worth noting that *GbYTH8* anchored on chromosome A10 in *G. barbadense* was not identified in *G. hirsutum*, indicating that duplication and deletion events may appear during evolution.

Polyploidy is one of the major mechanisms of plant formation and environmental adaptation [32]. Gene duplications are regarded as one of the key driving forces during the evolution of genomes and genetic systems. Duplicated genes offer raw material for the formation of novel genes, which may facilitate the generation of novel functions [33]. Tandem duplication, segmental duplication, and transposition events are three principal evolutionary patterns, of which tandem duplications and segmental duplications were found to be the main causes of gene family expansion in plants [34,35]. Furthermore, segmental duplications have been identified to play important roles in expanding the gene family members in plants [36,37]. In this study, no tandem duplication was detected in the *YTH* gene family, though the genes may have undergone independent evolutionary processes in two allotetraploids cotton according to the synteny analysis.

Environmental stress responses involve various physiological, cellular, and molecular adaptations. Plants can produce and accumulate phytohormones, such as abscisic acid (ABA), Jasmonic acid (JA), ethylene (ET), and salicylic acid (SA), in response to stresses [38]. Under stressed conditions, molecular level changes in plants are principally caused by transcription factors binding to the *cis*-regulatory elements upstream to the stress-responsive genes [39]. The pattern of existence of these *cis*-elements reveals the mechanism of stress-responsive upregulation of downstream genes [40]. In this study, many inducible elements, such as ABA, ET, and SA responsive elements were recognized in the promoter region of *YTH* genes, suggesting functions of cotton *YTH* genes in stress responses. Further research is required to decipher their roles in stress responses by recognizing their gene structures, protein structures, and target RNAs.

In general, the abundant or increased expression of a gene in a tissue, a developmental stage, or a stress condition may suggest its roles correlated to developmental and stress responses. Publicly available transcriptomic data were analyzed to explore the possible function of the *GhYTH* genes in development and stress responses [41]. The production and quality of cotton are closely related to the development of ovule and fiber. In our study, a set of *GhYTH* genes were expressed at higher levels during the development of the ovule and fiber of cotton. These highly expressed *YTH* genes in wheat spikes might function in the development and architecture of spikes [16]. Furthermore, the expressions of the *GhYTH* gene among different tissues were different, suggesting that they may play numerous roles in different cotton tissues. The *YTH* genes are known to play essential roles in stress responses in several plant species. In this present study, expressions of some *GhYTH* genes were induced after stress treatments. Similar expression patterns were also observed in *Arabidopsis*, rice, cucumber, and apple [14,20,21,42].

sRNAs (small RNAs) ranging from 21 to 24 nt have obtained much attention for their biological roles in plant growth, development, and biotic and abiotic stress responses [43]. MiRNAs are a sort of sRNA, which could suppress the expression of target genes via binding to the mRNAs [44]. Drought, one of the most severe abiotic stresses, limits crop growth and yield by changing metabolic activity and biological functions [45]. MiRNAs have been found to play important roles in the drought stress response in cotton. In this study, 18 miRNA targets of *GhYTH* genes were detected. *GhYTH3* and *GhYTH13* could be targeted by ghr-miR156a, ghr-miR156b, and ghr-miR156d. One of these targets, ghr-miR156a was expressed differently between control and drought treatment in a previous report [46]. In addition, the miR156 family was identified to serve as the main regulator in response to high-temperature stress during *G. hirsutum* anther development [47]. These predicted miRNA targets could provide valuable candidates for the experimental study of the stress response of *GhYTH* genes.

In humans, YTH domain-containing RNA-binding proteins were extensively studied [48]. YTHDF1/2/3 could bind to N6-methyladenosine and thus control the stability of mRNA [27]. YT521 (YTHDC1) is characterized by alternately spliced isoforms with regulatory impact on cancer [49]. In contrast, the functional research of the YTH proteins in plants is very limited. VIGS was widely used to explore the gene functions in cotton for the difficulties inherent in cotton transformation [50]. The effect of *GhYTH* silencing by VIGS was evaluated, and the results showed that the silencing of *GhYTH8* decreased the resistance of the plants to droughts. Our findings verified the *GhYTH* had a vital function in the resistance of *G. hirsutum* to droughts.

## 4. Materials and Methods

### 4.1. Identification and Physiochemical Properties Analysis of YTH Gene Family Members in Four Gossypium Species

The gene annotation and genome files of two diploid species, *G. arboreum* L. (CRI) and *G. raimondii* L. (JGI), and two tetraploid species, *G. hirsutum* L. (CRI) and *G. barbadense* L. (ZJU), were retrieved from the Cotton Functional Genomics Database (https://cottonfgd.org/(accessed on 11 January 2023)) [41]. The protein sequences of *YTH* genes in Arabidopsis were obtained from the TAIR10 database (https://www.arabidopsis.org/ (accessed on 11 January 2023)) [51]. The query sequences of candidate *YTH* genes against the protein sequences of four cotton species were aligned by BLASTp. The candidate genes were analyzed in the NCBI-CDD (https://www.ncbi.nlm.nih.gov/Structure/bwrpsb/bwrpsb.cgi (accessed on 11 January 2023)) database to identify the YTH domain. The identified *YTH* genes were further confirmed by HMM search function in HMMER3.0 [52]. The physiochemical properties, relative molecular mass (MW), and theoretical isoelectric point (pI) were determined through the ExPASy proteomics server (https://web.expasy.org/compute_pi/ (accessed on 11 January 2023)) [53]. The genomic locations of *YTH* genes in the chromosome were extracted from the annotation files and were visualized by MapChart (https://www.wur.nl/en/show/Mapchart/ (accessed on 11 January 2023)).

### 4.2. Evolutionary, Gene Structure, and Conserved Motif Analysis of YTH Genes

All the identified YTH protein sequences from four cotton species and Arabidopsis were aligned using MUSCLE algorithm program in MEGA-X [54]. The maximum likelihood (ML) method of MEGA-X was exploited to construct the phylogenetic tree with the LG + G model and 1000 bootstrap replications, followed by embellishing the phylogenetic tree with Evolview (https://evolgenius.info/ (accessed on 11 January 2023)) [55].

The conserved motifs of cotton YTH protein sequences were analyzed using the MEME program (http://meme-suite.org/ (accessed on 11 January 2023)) [56]. The gene structure information of the cotton *YTH* genes was retrieved from Cotton Functional Genomics Database, and the gene structures were graphically visualized using TBtools [57].

### 4.3. Chromosomal Distribution and Synteny Analysis of YTH Genes

CottonFGD genome annotation files were used to determine the chromosomal distribution of *YTH* genes in 4 cotton species. Duplication events and syntenic blocks of cotton *YTH* genes were found by McScanX [58], followed by visualizing the orthologous *YTH* genes between four cotton species through CIRCOS. Ka/Ks analysis was performed as previously described [59].

### 4.4. Regulatory Elements Analysis of YTH Genes

The 2000 bp upstream region of coding sequence (CDS) of *YTH* genes extracted using TBtools was regarded as the promoter region. The *cis*-acting regulatory elements of *YTH* genes were identified using PlantCare (http://bioinformatics.psb.ugent.be/webtools/plantcare/html/ (accessed on 11 January 2023)) [60] and then visualized by R.

### 4.5. Subcellular Localization of GhYTH8 and GhYTH16 Proteins

The full-length coding sequences of *GhYTH8* and *GhYTH16* were amplified using specific primers GhYTH8-GFP-F (5′ATACACCAAATCGACTCTAGAATGGCCACTCCTGATCGTAT-3′; an Xba I site underlined)/ GhYTH08-GFP-R (5′- CCCCTGCAGAAGCTTTCTAGAGCAAGCACTGGCTGCACACA-3′; an Xba I site underlined) and GhYTH16-GFP-F (5′-ATACACCAAATCGACTCTAGAAAAACCATCATACCCCAGCT -3′, an Xba I site underlined)/ GhYTH16-GFPR (5′-CCCCTGCAGAAGCTTTCTAGA ACCATGAGCTGTGCCCATGC-3′, an Xba I site underlined) and cloned into pCAMBIA1300-GFP. The recombinant plasmids, p1300 GhYTH8-GFP and p1300 GhYTH16-GFP, were transformed into *Agrobacterium tumefaciens* strain GV3101 and infiltrated into fully expanded leaves of *Nicotiana benthamiana* plants. GFP-expressing cells were detected with a confocal laser scanning microscope (Zeiss LSM 510 META; argon laser excitation wavelength, 488 nm).

### 4.6. Expression Profiles Analysis of YTH Genes

The transcriptional expression profiles of *YTH* genes in *G. hirsutum*, including ovule development (−3, 0, 1, 3, 5, 10, 20, and 25 DPA), fiber development (5, 10, 20, and 25 DPA), different tissues (torus, stem, sepal, root, pistil, petal, leaf, filament, bract, and anther), and different stresses (cold, hot, drought, and salt) were downloaded from CottonFGD. The value of the gene expression levels was processed with log2(FPKM + 1) and visualized by R.

### 4.7. MiRNA Prediction of GhYTH Genes

The targeted relationship between miRNA and *YTH* genes in *G. hirsutum* was predicted with psRNATarget (https://www.zhaolab.org/psRNATarget/ (accessed on 11 January 2023)) [61] and then visualized by Cytoscape-3.8.2 [62].

### 4.8. Virus-induced Gene Silencing (VIGS) Experiment and Drought Treatment

We performed VIGS experiments to further confirm whether *YTH* genes affect drought stress tolerance in cotton. Total RNA was extracted from the leaves and then reverse transcribed to cDNA. A 313 bp fragment of *GhYTH8* ORF was amplified and cloned into the pYL156 to construct the fusion vector pYL156:GhYTH8. The vectors (pYL156:GhYTH8 and pYL156:CLA1) were transformed into A. tumefaciens strain GV3101, followed by cultivating overnight in L.B. medium with kanamycin 50 mg/L and rifampicin 25 mg/L at 30 ℃. pYL156:00 (TRV:00), pYL156:CLA1, and pYL156:GhYTH8 were mixed with a 1:1 ratio, followed by agroinfiltrating into fully unfolded cotton cotyledons with a needle-free syringe. The standard variety TM-1 (*G. hirsutum*) was used as the experimental plant. After a night of darkness, the plants were transferred to a greenhouse at 20 °C with a 16 h light/8 h dark photoperiod. When TRV:CLA1 plants showed phenotype, TRV:GhYTH8 and TRV:00 plants were kept without water to simulate drought conditions.

### 4.9. Quantitative Real-Time PCR (qPCR)

Three sets of samples from different plants were collected as three biological replicates. The leaves of TRV:00 and TRV:GhYTH8 cotton seedlings were used for total RNA extraction with an EASYspin Plus Plant RNA Kit (Aidlab, Beijing, China) according to the manufacturer’s instructions. The RNA reverse-transcribed used Trans Script One-Step gDNA Removal and cDNA Synthesis SuperMix (Trans Gen, Beijing, China). Cotton Histon3 (GhHiston3) was used as an endogenous standard control. qPCR assays were performed on a QuantStudio 6 Flex thermocycler (Applied Biosystems, Foster City, CA, USA) using Trans Start Top Green qPCR SuperMix (TransGen, Beijing, China) and repeated three times.

## 5. Conclusions

Above all, our results highlight the potential role of the *YTH* genes that are involved in the drought stress response. Our results provide new insights to further study the biological roles of the *YTH* genes in stress response.

## Figures and Tables

**Table 1:** Characteristics of *YTH* genes and their encoded proteins in cotton.

Gene ID	Gene Name	Amino Acid Length	P.I.	MW (da)	Location
Ga01G0324.1	*GaYTH1*	654	6.19	72224.25	Chr01:2562085–2564534(-)
Ga02G0908.1	*GaYTH2*	625	6.83	68261.89	Chr02:60033655–60039884(-)
Ga03G0644.1	*GaYTH3*	654	5.83	72141.35	Chr03:9431937–9434567(-)
Ga03G2762.1	*GaYTH4*	572	6.08	63500.31	Chr03:135380859–135385216(-)
Ga08G0210.1	*GaYTH5*	635	7.22	70257.79	Chr08:1723259–1728028(-)
Ga08G2483.1	*GaYTH6*	577	5.9	63763.03	Chr08:124586293–124589173(+)
Ga10G3082.1	*GaYTH7*	648	5.32	71361.54	Chr10:129290100–129295676(-)
Ga11G0956.1	*GaYTH8*	677	5.56	74319.92	Chr11:16221680–16226718(+)
Ga11G0958.1	*GaYTH9*	605	6.15	66573.51	Chr11:16288205–16291213(+)
Ga13G1351.1	*GaYTH10*	596	6.2	65509.66	Chr13:83803610–83807221(-)
Gbar_A01G002830.1	*GbYTH1*	566	8.48	62324.52	A01:2568244–2572387(-)
Gbar_A01G015130.1	*GbYTH2*	686	6.56	75281.04	A01:91702309–91709506(+)
Gbar_A02G005740.2	*GbYTH3*	654	5.92	72218.39	A02:8400243–8404612(-)
Gbar_A03G023470.1	*GbYTH4*	572	5.96	63511.37	A03:105084247–105089973(-)
Gbar_A05G011330.1	*GbYTH5*	653	5.2	71651.11	A05:10378466–10385178(-)
Gbar_A08G001890.1	*GbYTH6*	666	6.09	73438.17	A08:1669319–1673433(-)
Gbar_A08G022910.1	*GbYTH7*	577	6.01	63687.94	A08:115214249–115217627(+)
Gbar_A10G000210.1	*GbYTH8*	650	5.32	71521.71	A10:226672–232918(+)
Gbar_A11G027540.5	*GbYTH9*	605	6.15	66528.45	A11:98904170–98908097(-)
Gbar_A11G027580.1	*GbYTH10*	612	5.96	66900.74	A11:98946062–98949702(-)
Gbar_A13G011910.1	*GbYTH11*	607	6.08	66295.32	A13:73997171–74001401(-)
Gbar_D01G003000.1	*GbYTH12*	654	7.22	72240.22	D01:2591629–2595737(-)
Gbar_D01G016180.1	*GbYTH13*	625	6.72	68475.31	D01:47823428–47830617(+)
Gbar_D02G006470.1	*GbYTH14*	689	8.27	76200.42	D02:8464041–8470880(-)
Gbar_D02G025310.1	*GbYTH15*	572	6.21	63284.11	D02:67385264–67390933(-)
Gbar_D05G011770.9	*GbYTH16*	660	5.04	72234.67	D05:9785369–9791757(-)
Gbar_D08G001930.1	*GbYTH17*	670	6	73907.66	D08:1553392–1557467(-)
Gbar_D08G023590.1	*GbYTH18*	559	6.81	62082.17	D08:61689230–61692672(+)
Gbar_D10G000170.2	*GbYTH19*	650	5.38	71582.77	D10:164189–170278(+)
Gbar_D11G035010.1	*GbYTH20*	622	5.72	68122.11	Scaffold118:164271–170796(+)
Gbar_D11G035040.1	*GbYTH21*	605	6.26	66560.48	Scaffold118:211996–217213(+)
Gbar_D13G011640.1	*GbYTH22*	606	6.08	66234.25	D13:33076872–33081145(-)
Gh_A01G032100.1	*GhYTH1*	654	6.19	72210.18	A01:2855623–2858072(-)
Gh_A01G173200.1	*GhYTH2*	625	6.55	68290.89	A01:91578115–91584668(+)
Gh_A02G065800.1	*GhYTH3*	654	6	72278.51	A02:8801670–8804658(-)
Gh_A03G245900.1	*GhYTH4*	720	8.25	81093.52	A03:109862488–109870541(-)
Gh_A05G102800.1	*GhYTH5*	651	5.2	71407.77	A05:10898607–10904838(-)
Gh_A08G019200.1	*GhYTH6*	666	6.09	73404.17	A08:1725549–1729279(-)
Gh_A08G241100.1	*GhYTH7*	568	6	62764.8	A08:120898607–120901810(+)
Gh_A11G291200.1	*GhYTH8*	605	6.15	66501.43	A11:105609984–105613426(-)
Gh_A11G291500.1	*GhYTH9*	612	5.96	66900.74	A11:105651056–105655185(-)
Gh_A13G121400.1	*GhYTH10*	607	6.08	66322.34	A13:73017791–73021817(-)
Gh_D01G029500.1	*GhYTH11*	656	7.22	72458.49	D01:2546081–2549106(-)
Gh_D01G171300.1	*GhYTH12*	625	6.72	68445.22	D01:49030052–49036273(+)
Gh_D02G069800.1	*GhYTH13*	654	6.05	71898.2	D02:8345891–8350344(-)
Gh_D02G261200.2	*GhYTH14*	572	6.21	63400.27	D02:70853189–70858785(-)
Gh_D05G115100.3	*GhYTH15*	652	5.08	71170.49	D05:9802311–9808568(-)
Gh_D08G018500.1	*GhYTH16*	583	7.24	64432.55	D08:1638425–1641033(-)
Gh_D08G232400.1	*GhYTH17*	577	6.41	64071.27	D08:64381471–64384820(+)
Gh_D10G001600.3	*GhYTH18*	650	5.25	71466.59	D10:196546–202609(+)
Gh_D11G291400.1	*GhYTH19*	605	6.26	66560.48	D11:60000676–60004152(-)
Gh_D11G291600.1	*GhYTH20*	613	5.86	67164.03	D11:60044437–60048569(-)
Gh_D13G123000.1	*GhYTH21*	606	6.12	66218.25	D13:36489013–36493188(-)
Gorai.002G031800.1	*GrYTH1*	654	6.9	72227.26	Chr02:2370851–2374924(-)
Gorai.002G182900.1	*GrYTH2*	625	6.49	68392.05	Chr02:48153196–48160671(+)
Gorai.004G020600.1	*GrYTH3*	670	6	73973.81	Chr04:1530886–1534693(-)
Gorai.004G244600.1	*GrYTH4*	599	6.41	66812.77	Chr04:58179219–58182629(+)
Gorai.005G070500.1	*GrYTH5*	654	5.96	71958.21	Chr05:7641980–7646032(-)
Gorai.005G265300.1	*GrYTH6*	572	6.21	63296.12	Chr05:63865504–63871029(-)
Gorai.007G288900.1	*GrYTH7*	605	6.26	66610.57	Chr07:49501978–49505697(-)
Gorai.007G289200.1	*GrYTH8*	622	5.69	68135.13	Chr07:49545172–49551745(-)
Gorai.009G120700.1	*GrYTH9*	654	5.14	71573.99	Chr09:8935247–8942392(-)
Gorai.011G001700.1	*GrYTH10*	648	5.37	71416.54	Chr11:161861–168102(+)
Gorai.013G123200.1	*GrYTH11*	606	6.12	66162.19	Chr13:31722245–31726598(-)

## Data Availability

The genome of four cotton species and RNA-seq data were downloaded from the CottonFGD website (https://cottonfgd.org/ (accessed on 11 January 2022)).

## Supplementary Materials

The following supporting information can be downloaded at: https://www.mdpi.com/article/10.3390/plants12051198/s1, Figure S1: Conserved domains in cotton YTHs; Table S1: Exon numbers of *GhYTH* genes; Table S2: Ka/Ks ratio of *YTH* homologous genes between *G. hirsutum* and *G. barbadense*; Table S3: Relationships between the miRNAs and *GhYTHs*; Table S4: Tissue expression profiles of *GhYTH* genes; Table S5: Expression profiles of *GhYTH* genes under different stress.
